# De Novo A-to-I RNA Editing Discovery in lncRNA

**DOI:** 10.3390/cancers12102959

**Published:** 2020-10-13

**Authors:** Domenico Alessandro Silvestris, Chiara Scopa, Sara Hanchi, Franco Locatelli, Angela Gallo

**Affiliations:** 1 RNA Editing Lab, Department of Hematology and Oncology and Cell and Gene Therapy, Bambino Gesù Children’s Hospital (OPBG), IRCCS, 00146 Rome, Italy; asilvestris@alice.it (D.A.S.); chiara.scopa@opbg.net (C.S.); sara.hanchi@opbg.net (S.H.); franco.locatelli@opbg.net (F.L.); 2 Department of Pediatrics, La Sapienza, University of Rome, 00100 Rome, Italy

**Keywords:** RNA editing, lncRNA, brain, glioblastoma

## Abstract

**Simple Summary:**

Long non-coding RNAs are emerging as key regulators of gene expression at both transcriptional and translational levels, and their alterations (in expression or sequence) are linked to tumorigenesis and tumor progression. RNA editing has the unique ability to change the RNA sequence without altering the integrity or sequence of genomic DNA, with adenosine to inosine (A-to-I) RNA editing being the most common event in humans. With the ability to change the genetic information after transcription, RNA editing is an essential player in the transcriptome and proteome enrichment; however, when deregulated, it can contribute to cell transformation. In this article, we performed the first deep de novo editing survey in lncRNA, demonstrating that RNA editing is a pervasive phenomenon involving lncRNAs important in the brain and brain cancer. Our study will open a new field of research in which the interplay between lncRNA and RNA editing can add novel insights into cancer.

**Abstract:**

Background: Adenosine to inosine (A-to-I) RNA editing is the most frequent editing event in humans. It converts adenosine to inosine in double-stranded RNA regions (in coding and non-coding RNAs) through the action of the adenosine deaminase acting on RNA (ADAR) enzymes. Long non-coding RNAs, particularly abundant in the brain, account for a large fraction of the human transcriptome, and their important regulatory role is becoming progressively evident in both normal and transformed cells. Results: Herein, we present a bioinformatic analysis to generate a comprehensive inosinome picture in long non-coding RNAs (lncRNAs), using an ad hoc index and searching for de novo editing events in the normal brain cortex as well as in glioblastoma, a highly aggressive human brain cancer. We discovered >10,000 new sites and 335 novel lncRNAs that undergo editing, never reported before. We found a generalized downregulation of editing at multiple lncRNA sites in glioblastoma samples when compared to the normal brain cortex. Conclusion: Overall, our study discloses a novel layer of complexity that controls lncRNAs in the brain and brain cancer.

## 1. Introduction

RNA editing is a post-transcriptional mechanism that modifies RNA nucleotides without changing the template genomic DNA [1,2]. In humans, the most common type of RNA editing involves the adenosine to inosine (A-to-I) nucleotide conversion and is catalyzed by the adenosine deaminases that act on dsRNA (ADARs) family of enzymes, with ADAR1 and ADAR2 present in all tissues, while the catalytically inactive ADAR3 is expressed almost only in the brain [3]. These enzymes act as homodimers and deaminate adenosines within double-stranded RNAs [4,5]. Since inosines are recognized as guanosines (G) by the splicing and translation machinery, A-to-I RNA editing can modify RNA splicing sites, induce amino acid substitutions, and alter dsRNA structures/folding [6]. In recent years, it has been estimated, from computational studies and massive sequencing of RNA, that over 4.7 million editing sites exist in the human transcriptome [7,8]. Indeed, A-to-I RNA editing can provide an additional layer of complexity to the transcriptome, increasing the proteome landscape and modulating the structure and function of several non-coding RNAs, such as microRNAs (miRNAs) and long non-coding RNAs (lncRNAs) [9,10]. LncRNAs are classified as RNA transcripts longer than 200 nucleotides and lacking significant protein-coding capacity [11]. They resemble mRNAs, as they are generally transcribed by RNA polymerase II, 5′ capped, 3′ polyadenylated, and often undergo splicing of multiple exons via canonical genomic splice motifs [12].

LncRNAs play key roles in various biological processes in cell physiology, including imprinting control, cell differentiation, immune response, and chromatin modification [13,14]. Dysregulation of lncRNAs has been found to be relevant for neurological, cardiovascular, and developmental disorders, as well as cancer [15]. In fact, growing evidence shows that lncRNAs drive important cancer phenotypes through their interactions with other cellular macromolecules, such as RNA, DNA, and protein [16]. To date, A-to-I RNA editing in lncRNAs has been poorly investigated and, despite recent studies reporting that RNA editing may impact the secondary structure of RNA and the lncRNA–miRNA interactions, the role of the edited lncRNAs remains largely unknown [9,17]. The de-regulation of ADAR-mediated RNA editing can promote cell transformation and tumor progression [18], and bioinformatic analyses combined with experimental studies have shown that ADAR-mediated editing patterns differ in normal and cancer tissues, including brain cancer, such as glioblastoma (GBM) [19,20,21]. Although RNA editing in glioblastoma has been intensely studied in both protein-coding and non-coding portions of the transcriptome (mainly miRNA) [22,23,24,25,26], the crosstalk between A-to-I RNA editing and lncRNA in this cancer has not been deeply investigated. Herein, for the first time, we present data on the lncRNA inosinome landscape as analyzed in both normal brain cortex and glioblastoma; in particular, we will show new data on lncRNA de novo editing call analysis.

## 2. Results

### 2.1. De Novo RNA Editing in Long Non-Coding RNAs

**Figure 1:**
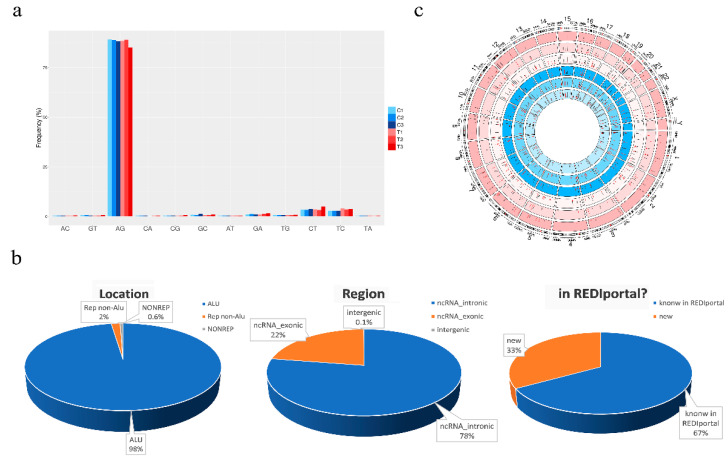
De novo RNA editing detection in long non-coding RNAs. (**a**) Bar graph shows nucleotide changes distribution. The plot indicates that most of the detected RNA editing events were A-to-G. All other changes were rare, with low substitution frequencies showing a high signal-to-noise ratio and specificity of our methodology. C1, C2, and C3 indicate the normal brain samples and T1, T2, and T3 the GBMs. (**b**) Pie charts showing the fraction of A-to-I sites discovered by our computational approach divided according to genomic location, gene region, and the presence or not in the REDIportal database (Table S1). (**c**) Circos plot reporting as black bars the A/G levels (%) distributed for each chromosome with the filter applied for a minimum coverage of 10 reads (data without filters are reported in Table S1). The analyzed samples are shown in concentric circles and ordered showing the three tumor tissues and the normal counterpart from the outside to the inside. Red bars indicate RNA editing sites specific for GBM or normal brain.

To deeply analyze the inosinome landscape of the lncRNA, we took advantage of a directional RNA-Seq dataset from three glioblastoma and matched normal brain tissues (cortex) [27]. The reads, after quality control and pre-processing steps, were aligned onto the reference human genome, and RNA editing candidates in lncRNAs were detected by using a modified version (see materials and methods) of a recently published pipeline [28]. The A-to-G mismatches identified by the above process are bona fide A-to-I RNA editing events, while the other types of mismatches provide an estimate of the false detection rate. The genome-wide screening conducted in three GBM samples and their normal controls yielded to high specificity (~90% of the nucleotide changes) of A-to-G changes with low noise, mainly due to T-to-C and C-to-T substitutions (Figure 1a). We reported a total of 31,267 potential A-to-I editing events, with 11,117 sites exclusively present in the normal brain cortex, 11,497 only present in GBM, and 8653 common sites (Table S1). According to another genome-wide computational screen [8], the majority of A-to-G/I changes (98%—30,555 unique A-to-G changes) were identified within *Alu* repeats and are particularly abundant (78%—24,256) in intronic regions of lncRNAs (Figure 1b). Additionally, the editing levels were depicted on chromosomes and shown by a Circos plot demonstrating the pervasive nature of the phenomenon (Figure 1c), with chromosomes 1 and X carrying a high number of editing sites (Figure 1c and Table S1). Of note, comparing our list of de novo edited positions with those already present in the REDIportal database, that includes also the positions of RADAR and DARNED databases [7,29,30], we found that 33% (10,445) of the identified editing sites are new positions that have never been reported before (Figure 1b and Table S1).

### 2.2. The Overall RNA Editing Level in lncRNAs is Reduced and Altered in GBM Versus Normal Brain

**Figure 2:**
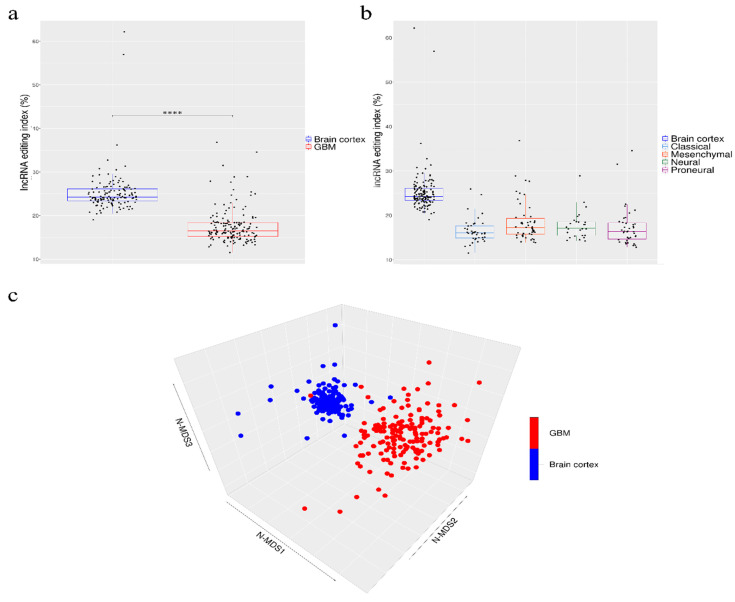
Overall amount of A-to-I RNA editing in long non-coding RNAs (**a**) Boxplots showing the distributions of long non-coding editing index values across normal brain cortex (132 samples) and primary glioblastomas (156 samples) and (**b**) glioblastoma subtypes. Two-tailed Mann–Whitney U test was applied. **** *p*  ≤  0.0001 (**c**) 3D-MDS (multidimensional scaling) analysis of RNA editing profiles in glioblastoma and normal cerebral cortex. Red and blue points indicate respectively GBMs and normal brains.

Our de novo editing sites list identified in lncRNAs was then utilized to screen a larger population of 156 primary glioblastomas and 132 normal cerebral cortex samples obtained respectively, upon authorization, from the TCGA dataset and from the GTEx project. In order to provide a suitable measure for the overall editing level at lncRNAs, we adapted the ‘recoding editing index’ [26] to calculate the average editing level at all the sites in lncRNAs. The ‘lnc-index’ is determined as the total number of reads with G at all editing positions in lncRNAs over the number of all reads covering the positions without imposing specific sequencing coverage filters. By applying this new metric, we found a very strong (*p*-value < 0.0001) global reduction of editing level in lncRNAs in glioblastoma compared to the normal brain (Figure 2a). When we compared the GBM samples stratified according to the well-known classification proposed by Verhaak [31], although there are small variations in the distribution of the lnc-index values, no statistically significant differences were observed (Figure 2b). Of note, the lnc-index values did not correlate with the expression level (transcripts per million (TPM)) of the *ADARs* transcripts in both GBM and normal brain (Figure S1). We performed a non-metric multidimensional scaling (MDS) analysis using Spearman’s correlation coefficients calculated by pairwise comparisons of RNA editing levels as identified in lncRNA, again without imposing any minimum coverage filter. As shown by the three-dimensional MDS graph, based on the editing profiles, two very clear clusters emerged representing the normal brain and GBM samples (Figure 2c), although some ‘outliers’ were observed. Of note, the cerebral cortex samples are grouped in a much more compact pattern than the tumor samples, confirming that there is an intra-tumoral heterogeneity in GBM, as also reported by several studies. We checked the possible presence, within the GBM population, of specific sub-clusters corresponding to the four subgroups from the Verhaak classification [31]; again, they are not evident from our analysis (Figure S2).

### 2.3. Discovery of Altered A-to-I Editing of lncRNA in Brain and GBM

To identify differentially edited sites within lncRNA in GBM and normal brain, we selected editing sites covered by at least 10 reads (with at least two reads supporting the variation) as detected in a minimum of 10 GBMs and 10 brain cortex samples and with Delta editing medians ≠ 0, thus obtaining a list of 1018 sites to test. We identified a total of 780 statistically significant differentially edited positions in GBM compared to cerebral cortex, as assayed by the two-tailed Mann–Whitney U-test followed by Benjamini–Hochberg multiple test corrections (Table S2). Of note, the statistical analysis approach utilized in this study was applied assuming that each single editing site behaves independently from the others.

**Figure 3:**
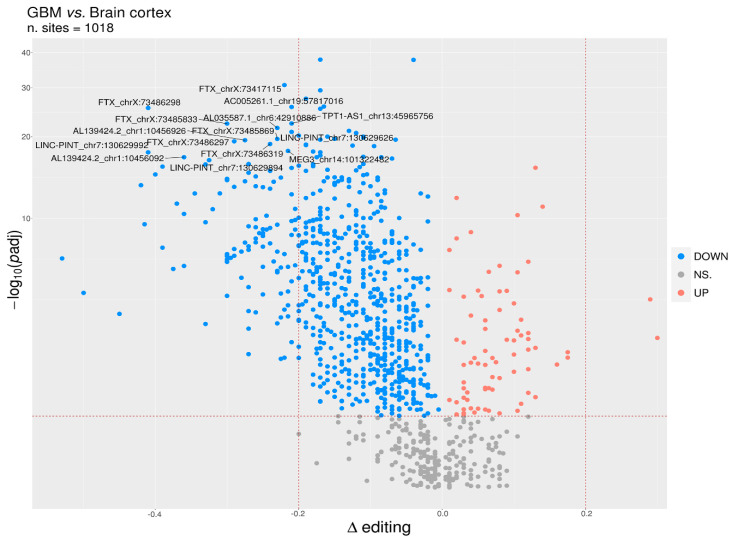
Identification of differentially edited sites in GBM. Volcano plot reporting the differentially edited sites between glioblastoma and normal cerebral cortex. The horizontal dotted line marks a multiple test-corrected level of significance (*padj* < 0.05, Mann–Whitney with Benjamini–Hochberg correction). *Y*-axis was reported in a “log1p” transformed scale. The vertical dotted lines indicate a Delta editing of 20% (0.2) and −20% (−0.2). Red, blue, and gray points indicate, respectively, over-edited (UP) sites, under-edited (DOWN) sites, and non-significative sites (NS.).

**Figure 4:**
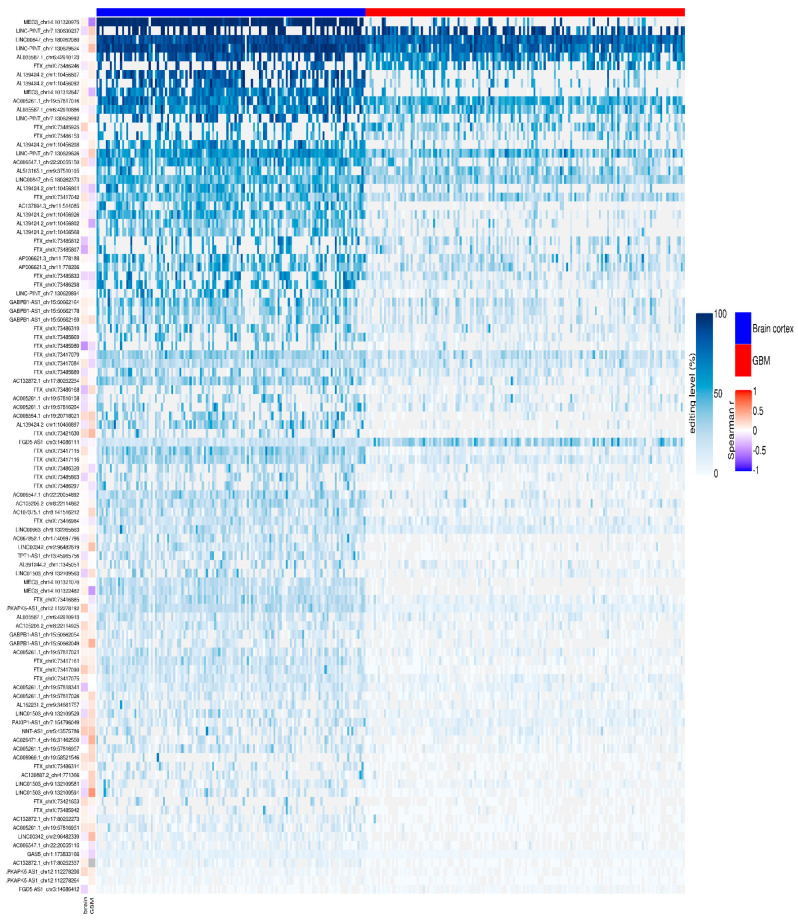
Editing levels at lncRNA sites. Heatmap with row clustering (dendrogram not shown) of editing levels (%) at the selected top 100 most relevant significantly differentially edited sites in glioblastoma compared to brain cortex. Each column represents one of the 156 de novo GBMs (red) and 132 brain cortex (blue) samples. In the vertical annotation bars on the left, for each editing site, the Spearman’s correlation coefficient of the editing level (%) and the gene expression level (TPM) is reported as calculated respectively by REDItools and StringTie.

We reported that 704/780 sites (90%) were under-edited in GMB, and 76 sites (10%) appeared over-edited in GBM (Figure 3 and Table S2). The editing levels of the 100 most significantly altered sites were also visualized by means of a heatmap plot (Figure 4) from which it is evident that, with a few exceptions, the vast majority of sites exhibit reduced editing levels in GBM compared to the normal brain. In addition, for each of these 100 differentially edited sites, we also calculated the Spearman’s correlation coefficient between the editing frequency and the expression of the transcript carrying the A/G substitution (TPM values) (Figure 4, left panel). We found that, despite generally low levels of significance, some sites showed an association (direct or inverted) in the brain and/or in GBM (Figure 4 and Table S3).

### 2.4. RNA Editing on FTX and MEG3 Transcripts

**Figure 5:**
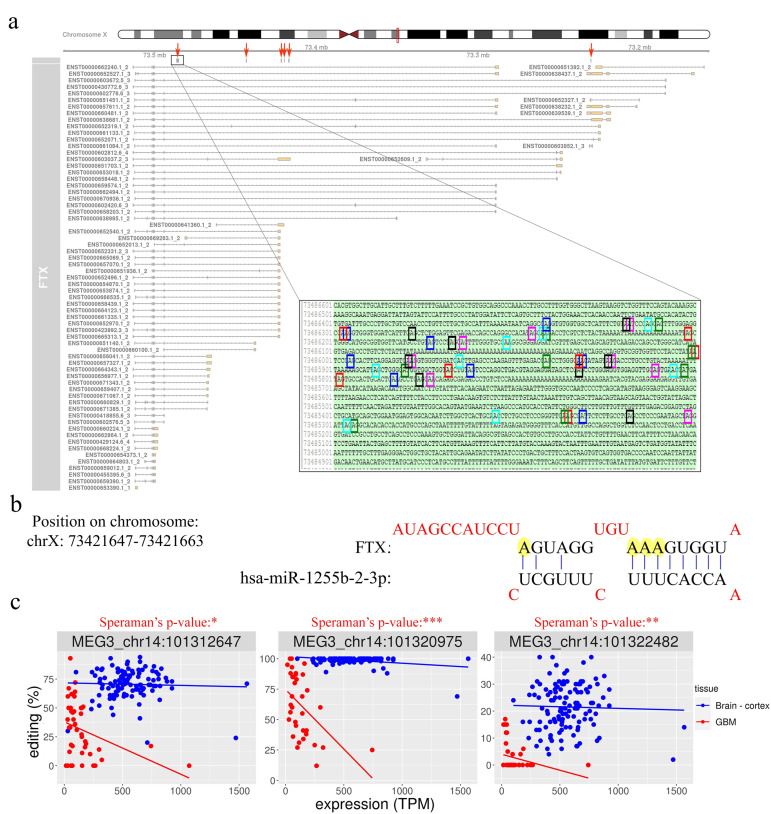
FTX long non-coding RNA is hyper-edited but downregulated in GBM. (**a**) Overview of FTX transcript structures as reported in the GENCODE/Ensembl annotation, with six red arrows indicating the clusters of editing sites that are significantly deregulated in GBM compared to normal brain cortex. In the box, an enlarged view of one of the edited adenosine clusters is shown at sequence level. (**b**) MicroRNA binding prediction on FTX transcript showing that some edited adenosines may potentially affect the miRNA–lncRNA interaction. (**c**) Correlation plots between MEG3 editing levels (%) and gene expression (TPM) for three sites differentially edited among GBM and normal cerebral cortex. Scatter plots and regression lines are reported in blue for normal brain samples and in red for GBMs. For each site, the Spearman’s correlation *p*-value calculated in GBM is also reported. * *p*  ≤  0.05, ** *p*  ≤  0.01, *** *p*  ≤  0.001.

Several very interesting lncRNA genes emerged from our differential editing analysis, carrying many A-to-I editing sites significantly dysregulated in GBM, some of which, according to the Mann–Whitney test, displayed high significance levels (e.g., LINC-PINT, FTX, or MEG3). Among all these genes, the FTX lncRNA, involved the X-inactivation center region [32], is particularly impressive with 91 differently edited sites organized in six clusters (Figure 5a), and with several newly identified editing sites (Table S2). Considering that many lncRNA exerted their function acting as a ‘sponge’ for miRNAs [11], we tested whether the editing sites in FTX may lay in miRNA binding regions. To predict the miRNA–lncRNA interactions, we used ‘LncBase Predicted v.2′ [33], which is part of the DIANA tools, and for each of the 91 sites deregulated in GBM we checked the possibility of binding a miRNA near the editing site. Interestingly, for eight editing sites (all localized within the third cluster in Figure 5a), a possible binding between FTX lncRNA and one or more miRNAs was predicted (Table S4). Specifically, we report that chrX: 73,421,817 and chrX: 73,421,630 editing sites can alter the binding of multiple miRNAs (Table S4). Of note, these two editing sites can fall into both an intronic and an exonic portion depending on the FTX transcribed isoform (see the third cluster/red arrow from the left in Figure 5a). Indeed, according to most FTX transcripts, these sites lay within introns, yet a small number of poorly supported transcripts suggest that they could lie in an exon in some cases. We also found that four closer editing sites lay in the predicted binding site for miR-1255b-2-3p (Figure 5b and Table S4). Another intriguing possibility, which needs to be studied further, is that these editing sites influence the shape and stability of the secondary structure of the lncRNA FTX and therefore indirectly affect its biological function.

Another long non-coding RNA on which we have focused our attention is MEG3. We found that, similar to FTX, MEG3 is also characterized by the presence of many (11) sites differentially edited in GBM compared to normal brain (Table S2). All the differentially edited sites are deregulated, with an under-editing trend in glioblastoma, and editing levels range from 99% for a site in the normal brain to 0% for some positions in the GBM. Interestingly, a direct correlation of editing and expression level was found at three sites in glioblastoma, according to the Spearman’s test (Table S3 and Figure 5c). Even more intriguing is the result of the binding prediction for miRNAs in regions of MEG3 corresponding to the edited adenosines. We found that one of the most significantly differentially edited sites is potentially targeted (in its unedited form) by six different miRNAs (Table S4).

## 3. Discussion

Recent evidence indicated lncRNAs as versatile molecules capable of acting as oncogenes or tumor suppressors in various tumors, including gliomas. LncRNAs are abundantly expressed in the brain, even if, for the vast majority of them, no role has been functionally characterized.

RNA-Seq and bioinformatic approaches have disclosed the pervasive presence of A-to-I RNA editing in the human transcriptome, including mRNA, miRNA, and lncRNA [34,35,36], but the role played by RNA editing in lncRNA is still under study. A recent study indicates that ADAR may contribute to the modulation of PRUNE2 level upon editing events within the dsRNA structure formed between PRUNE2 and the lncRNA PCA3 [37].

Although A-to-I editing sites have been identified in some lncRNAs, as mentioned above, an exhaustive editing survey in lncRNA has never been attempted. Herein, we applied a new metric for an ad hoc call of editing events in lncRNA, and we performed a de novo editing search within lncRNAs in both normal human brain cortex and GBM. We identified >10,000 novel editing sites and 335 novel lncRNAs undergoing editing that have never been reported before. Moreover, we found that editing signature in lncRNAs can clearly separate GBM and normal brain samples, as shown by the MDS analysis. Comparing the inosinome fingerprint of normal brain and GBM samples, a notable difference between the two sample tissues emerged, with an overall higher editing level in normal brain cortex compared with GBM samples. The editing decrease in GBM in lncRNAs is similar to what was previously reported in whole transcriptome analyses [25,26].

We also reported that a few highly edited lncRNA sites are present in tumor samples compared to the normal brain, with only two lncRNAs (AC139795.2 and AC006511.7) displaying an editing increase >20% in GBM (Delta >−20%, Table S2). No additional information is available regarding the role exerted by these two lncRNAs.

Among the highly edited lncRNAs in the normal brain cortex, there are FTX and MEG3. LncRNA FTX was firstly identified in the Xist gene locus, and it is one of the lncRNAs that takes part in the X-chromosome inactivation, as it can positively regulate Xist [38]. Interestingly, we found that important lncRNA genes involved in the X-chromosome inactivation (such as FTX, Xist, and JPX) all undergo extensive editing, opening the fascinating possibility that the RNA editing machinery can play a role in this sophisticated and still not completely understood mechanism. The lncRNA FTX is particularly unique among the edited lncRNAs, with >90 differently edited sites in normal brain and GBM, most of them identified herein. Of note, editing events within FTX are organized in six distinct clusters. Interestingly, FTX promotes glioma proliferation and invasion through the binding of specific miRNA [39], and as the editing can alter the miRNA binding ability on RNA targets [40], we searched for the miRNA–lncRNA interactions. FTX can bind multiple miRNAs at sites undergoing editing; this finding suggests that ADARs can modulate the binding ability of this lncRNA. A recent study identified FTX as an oncogenic factor in GBM that increases during radiation exposure together with NEAT1, and both these lncRNAs are involved in cancer radio-resistance [41]. Herein, we add another piece of information indicating that both NEAT1 and FTX are highly edited at multiple sites (Table S1), with the FTX transcript showing significantly decreased editing levels in GBM samples compared to controls. Overall, considering the emerging roles played by FTX in cancer, we believe that our finding can shed new light on the regulation of this lncRNA in GBM.

Together with FTX, the maternally expressed imprinted long non-coding RNA MEG3 was also found to be highly edited in the normal brain cortex. MEG3 is highly expressed in normal human tissue, but its expression is either decreased or abolished in many cancers, including GBM [12]. Indeed, it has been shown that low expression of MEG3 correlates with short survival in GBM patients and, if reintroduced in glioma stem cells (GSCs), it inhibits cell proliferation and in vivo tumor growth [42]. Downregulation of MEG3 is thought to be caused, at least in part, by the hyper- methylation of the MEG3 promoter region. Here, we identified 11 editing sites that were significantly and differently edited in GBM and normal brain, with editing that significantly decreased in GBM. More importantly we found a significant direct correlation between editing event and MEG3 level of expression in GBM, indicating that loss of editing at these sites is linked to the decreased MEG3 expression (Figure 5c). These findings open the possibility that editing may be an alternative mechanism for modulating MEG3 expression. Additionally, MEG3 can act in multiple cancer types as a ‘sponge’ of various miRNAs, thereby inhibiting the promotion of cancer phenotypes. MEG3 can bind a few miRNAs at the sites that undergo editing (among them, miR-939-39 and miR-331-3p). Interestingly, miR-331-3p can inhibit glioma progression [43], while miR-939-3p can promotes hepatocellular carcinoma and lung cancer [44,45]. We believe that editing loss in lncRNA in GBM can play an important role in either releasing miRNAs that can promote cancer (miR-939-3p) and/or binding tumor suppressor miRNAs (miR-331-3p).

Overall, our data demonstrated that lncRNAs can undergo extensive editing in normal brain and that the editing-lncRNA landscape is modulated in GBM. We show that the inosinome can superimpose another layer of information in lncRNAs, indicating, once more, that the ADAR enzymes are essential players in brain and glioblastoma.

## 4. Materials and Methods

### 4.1. Data

RNA-Seq data (total RNA) for de novo RNA editing discovery were obtained as FASTQ files from the NCBI SRA repository (SRP083311). Libraries were strand-specific and deeply sequenced with Illumina HiSeq2500 in three biological replicates, generating a total of 28.64 million paired-end 150-bp sequencing reads. For differential editing analysis, normal brain controls (132 cerebral cortex), with on average 39 million reads (paired ends) per sample, were downloaded from the Genotype-Tissue Expression (GTEx), and primary GBMs (156 samples), with on average 48 million reads (paired ends) per sample, were downloaded from The Cancer Genome Atlas (TCGA). Both datasets, with the same read length (76 bp) and generated from polyA-selected RNA, were downloaded upon authorization from the database of Genotypes and Phenotypes (dbGaP) with accession numbers phs000424.v8.p2 and phs000178.v11.p8, respectively.

### 4.2. Quality Check and Genome Mapping of RNA-Seq Data

RNA-Seq reads in FASTQ format were inspected using FASTQC program. Adaptors and low quality regions (phred cutoff of 20 for at least 70% of the read length) were trimmed using FASTP (0.20.1) [46], excluding reads with final lengths less than 55 bases. Cleaned reads were subsequently aligned onto the complete GRCh37/hg19 human genome by means of HISAT2 2.2.0 [47] (with very sensitive parameters), providing a non-redundant collection of known splice sites extracted from RefSeq and GENCODE databases. We also included dbSNP Common 151 in the index in order to take into account, during the mapping, the genetic variability of individuals, thus obtaining on average 98% alignment rates. Paired and concordant alignments in SAM format were converted in the binary BAM format by SAMtools, and duplicated reads were marked using the Picard MarkDuplicates.jar (2.21.9) tool (https://broadinstitute.github.io/picard/).

### 4.3. RNA Editing Analysis

RNA editing candidates in lncRNAs were called using the REDItoolDnaRna.py script. The first line of command of the REDItools package (-s 2 -g 2 -S -m 60,60 -q 30,30 -d -e -c 1,1 -n 0.0 -v 1 -p -u -l -z) is reported. LncRNA transcript annotation was downloaded from GENCODE 34 database (GRCh37 version including 18,051 entries). Following the detection scheme as in the protocol published by Lo Giudice and colleagues [28], initially nucleotide changes were called using loose parameters, then, in the absence of matching DNA information, all currently known single nucleotide polymorphisms (SNPs) and all somatic mutations identified to date in GBM were excluded. For each table, we separated positions residing in Alu elements, repetitive non-Alu regions, and non-repetitive regions, and for RNA editing candidates in repetitive non-Alu regions and non-repetitive regions more stringent filters were applied. In addition, reads supporting variants were collected and mapped onto the reference genome using PBLAT [48] to detect reads mapping on multiple genome locations with similar scores. In addition to the normal filters, all the positions overlapping non-lncRNAs transcripts were excluded by crossing their genomic coordinates with ‘bedtools intersect’, so as to obtain a more genuine and reliable signal of A-to-I editing in lncRNAs. All the edited positions thus collected for each of the three GBM samples and matching normal controls were merged in a comprehensive and non-redundant list of RNA editing events that was finally annotated by ANNOVAR. Our lnc-RNA editing list was used to interrogate the larger TCGA primary GBM and GTEx normal cerebral cortex datasets in order to compare the lnc-inosinome profiles and perform differential editing analysis.

### 4.4. Gene Expression

Gene expression levels were calculated in transcripts per million (TPM) units using StringTie 2.1.3 [49] and GENCODE annotation (release 34 back-mapped to the GRCh37 assembly).

### 4.5. Statistical Analyses and Plots

Differential editing statistics were calculated with SciPy Python library. Multidimensional scaling (MDS) was carried out in R using the ‘metaMDS’ function of the vegan package, providing as input a Spearman correlation matrix calculated from editing levels for each sample without imposing coverage filters. Plots were generated in R with ggplot2, circlize, ComplexHeatmap, and ggrepel libraries.

## 5. Conclusions

Herein, we showed the first exhaustive survey of a de novo RNA editing call in lncRNAs in normal brain cortex and glioblastoma. We demonstrated that lncRNAs, among them some oncogenes and tumor suppressor lncRNAs, are deeply modified by A-to-I RNA editing machinery, with editing that is significantly altered in cancer cells.

## Supplementary Materials

The following are available online at https://www.mdpi.com/2072-6694/12/10/2959/s1: Figure S1: Correlation between the expression of ADAR1 and ADAR2 (TPM) and lnc-editing index (%) in Brain cortex and GBM, Figure S2: Lnc-editing based non-metric three-dimensional scaling of the brain cortex and GBM samples divided by subtypes according to Verhaak classification. Table S1: De novo editing sites in three samples of glioblastoma and their corresponding normal tissues, Table S2: Differential editing analysis of brain-cortex (132) and GBM (156) samples, Table S3: Editing—expression correlation in brain-cortex and GBM, Table S4: miRNA-lncRNA interactions prediction by lncBase v2.
