# Supplementary figures and images for: De Novo A-to-I RNA Editing Discovery in lncRNA
Source: Cancers (Basel). 2020 Oct 13;12(10):2959. doi: 10.3390/cancers12102959 (PMC7650826; doi:10.3390/cancers12102959)

a

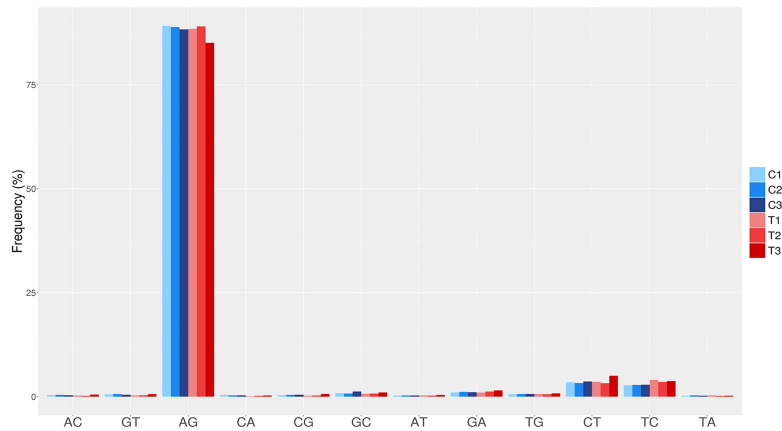

b

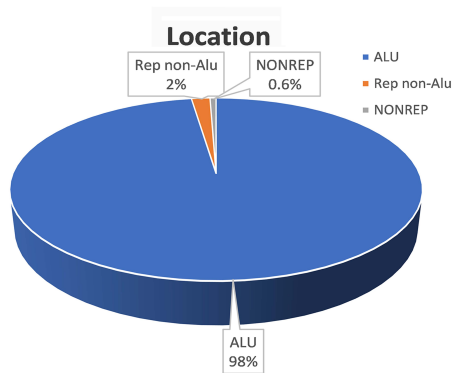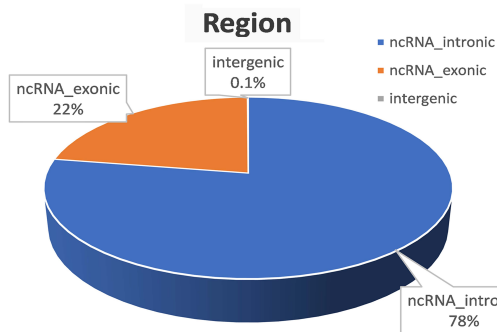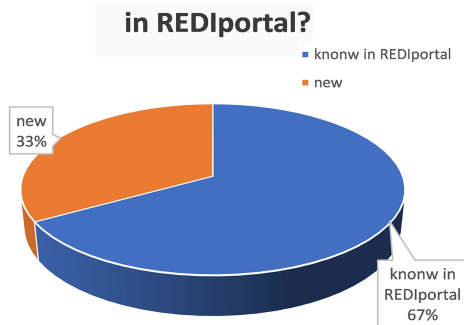

c

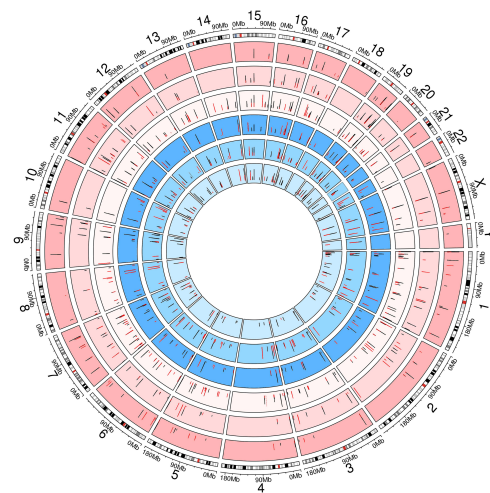

Supplement: Supplementary file 1 [file cancers-12-02959-s001.zip › cancers-916762-supplementary/cancers-916762-Figure 1 update.pdf]

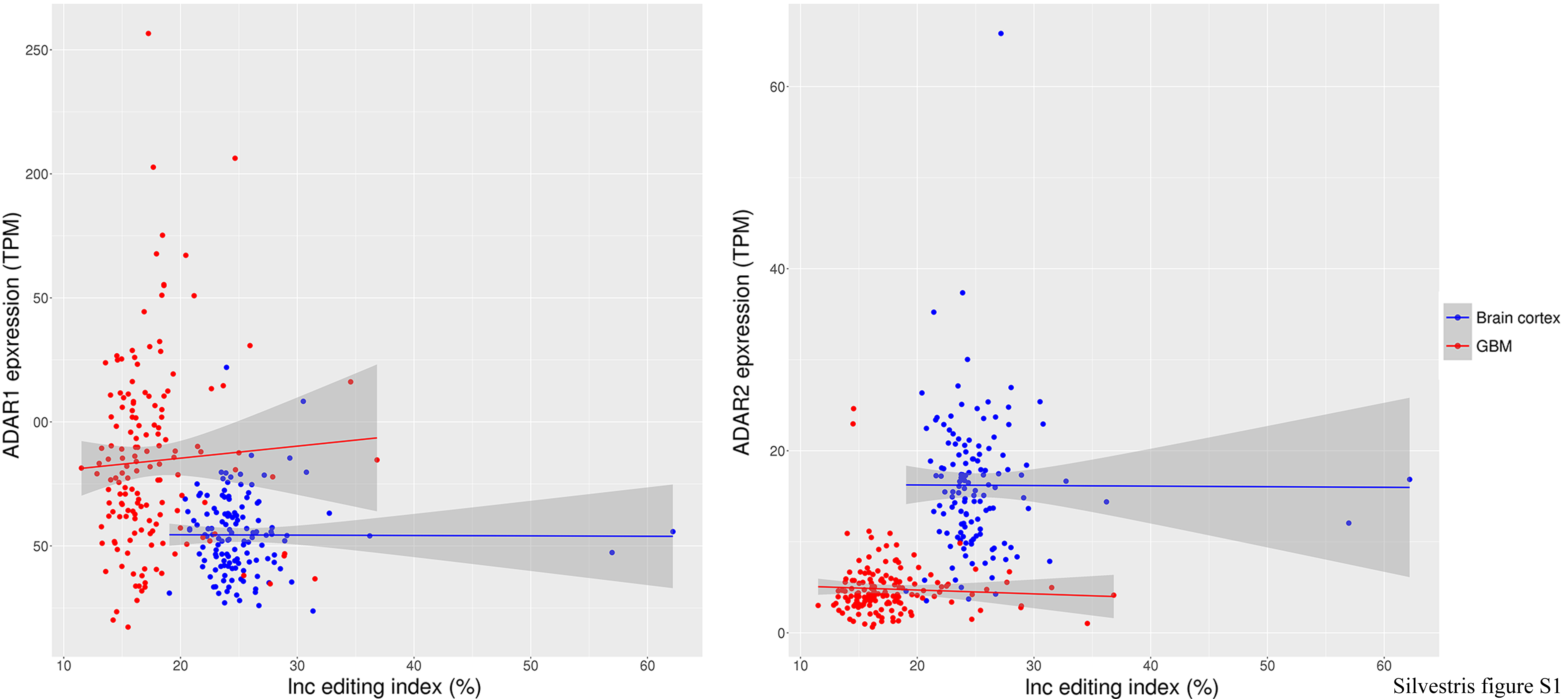

Supplement: Supplementary file 1 [file cancers-12-02959-s001.zip › cancers-916762-supplementary/Figure S1.tif]

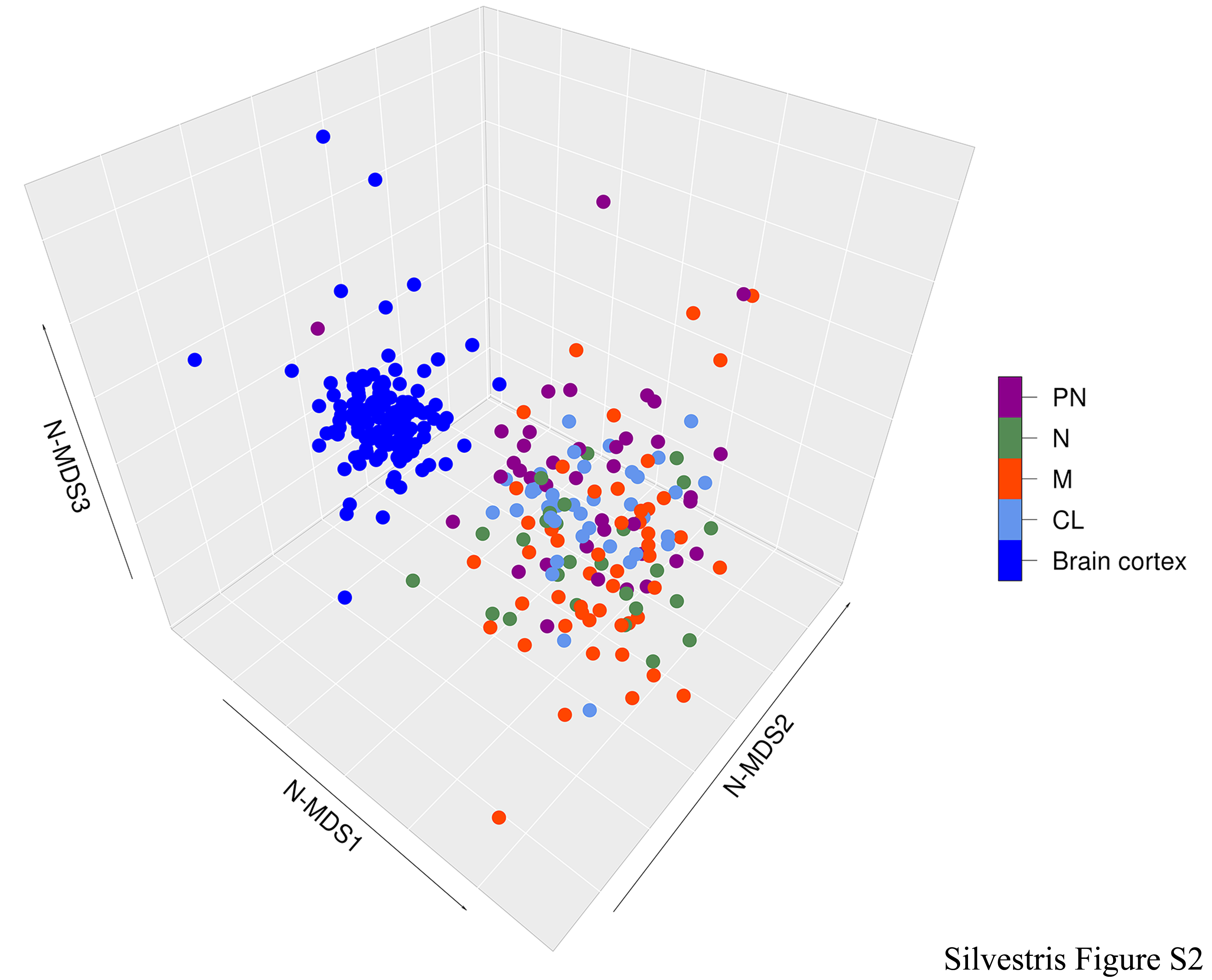

Supplement: Supplementary file 1 [file cancers-12-02959-s001.zip › cancers-916762-supplementary/Figure S2.tif]
